# Is Hepatitis B Immunoglobulin Necessary in Prophylaxis of Hepatitis B Recurrence after Liver Transplantation? A Meta-Analysis

**DOI:** 10.1371/journal.pone.0104480

**Published:** 2014-08-07

**Authors:** Peijie Wang, Ngalei Tam, Haochen Wang, Huanwei Zheng, Philip Chen, Linwei Wu, Xiaoshun He

**Affiliations:** 1 Organ Transplant Center, First Affiliated Hospital, Sun Yat-Sen University, Guangzhou, China; 2 Zhongshan School of Medicine, Sun Yat-Sen University, Guangzhou, Guangdong, China; 3 Hepatobiliary Surgery Department, the University of Hong Kong - Shenzhen Hospital, Shenzhen, Guangdong, China; 4 Liver Disease Department, Shijiazhuang Fifth Hospital, Shijiazhuang, Hebei, China; 5 Clinical Center for Liver Disease, University of Texas, Southwestern Medical Center, Dallas, Texas, United States of America; 6 Nephrology Department, University of Texas, Southwestern Medical Center, Dallas, Texas, United States of America; The University of Hong Kong, Hong Kong

## Abstract

**Background & Aims:**

Application of nucleoside analogues and hepatitis B immunoglobulin (HBIG) has reduced hepatitis B virus (HBV) recurrence rate after liver transplantation (LT) dramatically. Recent data suggests therapy without HBIG is also effective. We sought to evaluate the necessity of HBIG in prophylaxis of HBV recurrence after LT.

**Methods:**

A meta-analysis was performed. PubMed/MEDLINE, Web of Knowledge and other databases were searched for eligible literatures. The major end points were recurrence rate, patient survival, and YMDD mutant. Risk difference (RD) or risk ratio (RR) was calculated to synthesize the results.

**Results:**

Nineteen studies with a total of 1484 patients were included in this analysis. Application of HBIG was helpful to reduce HBV recurrence [P<0.001; RD = 0.16; 95% confidence interval (CI)(0.12, 0.20)] and virus mutants [P<0.001; RR = 3.13; 95%CI (1.86–5.26)], it also improved patients' 1-year [P = 0.03; RD = 0.08; 95%CI (0.01, 0.15)] and 3-year survival rates [P = 0.005; RD = 0.17; 95%CI(0.05, 0.28)]. No significant difference was found for patients' 5-year survival [P = 0.46; RD = −0.06; 95%CI (−0.21, 0.10)]. Sub-group analysis showed that in patients with positive pre-operative HBV DNA status, HBIG was necessary to reduce HBV recurrence rate (P<0.001; RD = 0.42; 95%CI (0.32, 0.52)). In patients with negative HBV DNA, combined therapy gained no significant advantages (P = 0.18; RD = 0.06; 95%CI (−0.03, 0.14)). Non-Lamivudine (non-LAM) antiviral drugs performed as well as combination therapy in prophylaxis of HBV recurrence after LT (P = 0.37; RD = 0.06; 95%CI (−0.02, 0.14)).

**Conclusions:**

HBIG with nucleoside analogues is helpful to reduce HBV recurrence and virus mutants. The necessity of HBIG in prophylaxis of HBV recurrence after LT when using new potent nucleoside analogues, especially for patients with negative pre-transplant HBV DNA status remains to be evaluated.

## Introduction

Over 400 million people have been infected with chronic hepatitis B virus (HBV) worldwide, with two-thirds of them in Asia [1]. End-stage HBV related liver diseases, including hepatic cirrhosis, liver failure, and hepatocellular carcinoma, are major indications of liver transplantation (LT) in the above area [2]. However, recipients might suffer from HBV recurrence after LT [3], [4], [5], [6]. In patients without any prophylaxis, HBV recurrence rate can reach as high as 80% [3], [4], [5]. The application of the first nucleoside analogue, lamivudine (LAM), reduced the recurrence rate of hepatitis B virus after LT dramatically. Unfortunately, its long-term use was associated with the risk of YMDD mutants, which would lead to the failure of hepatitis prevention, and possibly even the loss of the graft and the death of the recipient [7], [8], [9]. Hepatitis B immunoglobulin (HBIG) is efficient as a passive immune agent against HBV. Long-term passive immunoprophylaxis after LT results in a 60–80% reduction of HBV recurrence [10]. The combination of antiviral drugs and HBIG significantly reduced HBV recurrence rate and YMDD mutants; this strategy is also widely accepted as a routine prophylaxis for HBV recurrence after LT [11], [12], [13], [14].

With the application of new potent nucleoside drugs, some studies have illustrated the effectiveness of nucleosides without HBIG, not only for preventing HBV recurrence but also for controlling YMDD mutants [15], [16], [17], [18], [19]. Considering the inconvenience and high cost of long-term HBIG usage as well as the surveillance of hepatitis B surface antibody (HBsAb), the strategy without HBIG would be advantageous if could achieve the same effect. Some analysis have been conducted to compare the efficacy of LAM and HBIG combination therapy with that of LAM monotherapy [11]–[14], the previous studies have proven the advantages of combined therapy, but the role of HBIG in the era of new nucleosides remains unknown. To gain a better insight into this issue, we performed this meta-analysis to determine the necessity of HBIG in prophylaxis of HBV recurrence after LT. In addition to the observations described by the previous analysis, we also focused on the application of new nucleotides antiviral drugs and of the influence of patients' pre-transplant HBV DNA status.

## Patients and Methods

### Search Strategy

The primary aim of this meta-analysis was to compare the efficacy of antiviral drug therapy with that of antiviral drugs plus HBIG combination therapy after LT. We searched PubMed, Web of Knowledge databases, and Chinese databases including CNKI, Wan Fang and SinoMed until July 2013 to find human studies published. Regardless of language, key words used in the electronic search included ‘liver transplantation’ ‘hepatitis B’ ‘recurrence’ ‘HBIG’ ‘antiviral drugs’. In addition, we reviewed the reference lists of retrieved papers and recent reviews. Hepatitis B recurrence was defined as persistence of HBsAg for 3 weeks, as well as its reappearance in serum after LT.

### Inclusion and Exclusion Criteria

We set the following inclusion criteria for the studies: (1) Prospective or retrospective cohort studies investigating patients with LT; (2) Studies in which a comparison between antivirals therapy and combination therapy was designated as a primary aim; (3) studies providing sufficient description of the methods; and (4) studies reporting sufficient data on one of the following results: patients' survival, hepatitis B recurrence rate and YMDD mutants.

The following types of studies were excluded from our analysis: (1) unrelated or in vitro studies; (2) case series, case reports, reviews and conference reports; and (3) studies based on overlapping cohorts from the same institution;

When results from the same center were reported more than once, the newest was extracted. When results from some or all patients in a clinical trial were reported more than once, data on endpoints from the publication with the longest follow-up were extracted.

### Quality Assessment

A modified Methodological Index for Non-randomized Studies (MINORS) [20] was used to assess the quality of all trials included in this meta-analysis. All of the included trials were assessed for their study aims, patients, data collection, follow-up, groups' characteristics and statistical analyses.

### Statistical Analysis

We reviewed all the reported studies. Data were extracted according to clinical and statistical characteristics, duration of follow-up, HBV recurrence, YMDD mutants, and patient survival rate (1 year/3 years/5 years). All of these parameters were analyzed by using the Review Manager Software (version 5.0 for Windows; the Cochrane Collaboration, Oxford, UK), and the results were expressed by risk ratio (RR) or risk difference (RD) with 95% confidence intervals (CI).

## Results

### Search

**Figure 1:**
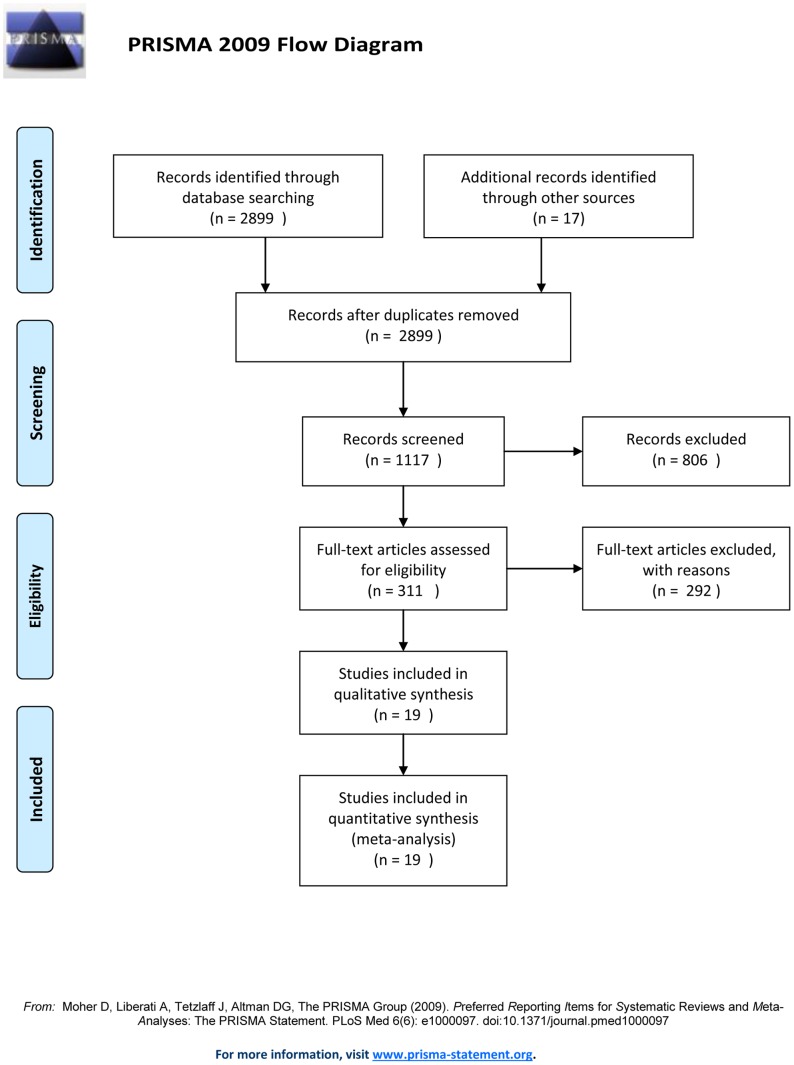
Literature search and selection flow: an overview of the methods used during the literature search.

An initial electronic search identified 2899 reports. After the first review, 1782 were excluded for lack of relevance of title or abstract. Among the remaining 1117 articles, 806 were excluded for unrelated trials' design, and 67 for lacking data on antiviral drugs therapy and antiviral drugs plus HBIG therapy. So, there were 244 reports for more detailed evaluation. After the second review about HBV recurrence, patient survival and YMDD mutant, there were 19 reports determined to be included in the meta-analysis. In total, there are 1484 patients included in this meta-analysis, with 484 in the antivirals therapy group and 1000 in the combination therapy group (Fig. 1).

### Trial Characteristics

Baseline features of the trials included are shown in Table 1. All the 19 studies included antiviral drugs group and combination therapy group. Study years and follow-ups are not stated in 2 studies. The other 17 had complete follow-up. Patients covered by 17 articles had a follow-up of 6–83 months with different emphases on HBV recurrence, YMDD mutants or patient survivals. Of the 19 included studies, 14 administered LAM antivirals therapy in one group and LAM+HBIG combination therapy in the other group. Among the other 5 studies, adefovir dipivoxil (ADV) (10 mg per day) combined with LAM were used in 3, ADV monotherapy was used in 1, and emtricitabine (FTC) (200 mg per day) with tenofovir disoproxil fumarate (TDF) (300 mg per day) in 1. Among these studies, the dosage of LAM is 100 or 150 mg/day and the average dosage of HBIG are 1000 units per month. Quality of included trials was assessed based on the Methodological Index for Non-randomized Studies (MINORS) [20](Table 2).

**Table 1:** Characteristics of trials included*

Study	Location	Study years	Follow-up (months)	No. patients	Antiviral drugs group(No. patients)	Combination therapy group(No. patients)
Buti et al. 2007 [21]	Spain	1998–2000	83	29	LAM(n = 20)	LAM+HBIG(n = 9)
Chun-Hui Yuan 2013 [22]	China	2000–2011	47.2	22	LAM(n = 6)	LAM+HBIG(n = 16)
Chung Mau Lo 2005 [23]	Hong Kong, China	1999–2004	21.1	16	LAM/ADV(n = 8)	LAM+ADV+HBIG(n = 8)
Dai J 2009 [24]	China	Not mentioned	69.14	55	LAM(n = 13)	LAM+HBIG(n = 42)
Dean M. Anselmo 2002 [25]	USA	1984–2001	29	109	LAM(n = 20)	LAM+HBIG(n = 89)
Dennis A. Freshwater 2008 [12]	UK	Not mentioned	Not mentioned	40	LAM(n = 10)	LAM+HBIG(n = 24)
LAM+ADV+HBIG(n = 6)						
Lewis W. Teperman 2013 [26]	USA	2007–2011	72	37	FTC/TDF(n = 18)	FTC/TDF+HBIG(n = 19)
Ma Y 2009 [27]	China	2001–2007	33.6	316	LAM(n = 106)	LAM+HBIG(n = 210)
Peter W. Angus 2008 [28]	Australia & New Zealand	2004–2006	21.1	34	ADV/LAM(n = 16)	LAM+HBIG(n = 18)
Shusen Zheng 2006 [29]	China	1999–2004	20.13	165	LAM(n = 51)	LAM+HBIG(n = 114)
Xia J 2007 [30]	China	1999–2004	44	98	LAM(n = 40)	LAM+HBIG(n = 58)
Xia N X 2006 [31]	China	2002–2004	18	173	LAM/ADV(n = 5)	LAM/ADV+HBIG(n = 168)
Xia Q 2004 [32]	China	2001–2003	6	58	LAM(n = 15)	LAM+HBIG(n = 43)
Yoshida H 2007 [33]	USA	1994–2004	67, 54 for two groups	60	LAM(n = 26)	LAM+HBIG(n = 34)
Yuan G Y 2002 [34]	China	Not mentioned	Not mentioned	15	LAM(n = 13)	LAM+HBIG(n = 2)
Jiao ZY 2007 [35]	China	1999–2005	37	84	LAM(n = 28)	LAM+HBIG(n = 56)
Zhu JP 2003 [36]	China	2000–2001	9.3	24	LAM(n = 15)	LAM+HBIG(n = 9)
Schiff 2007 [37]	Multi-center	1999–2003	7.7	57	ADV(n = 23)	ADV+HBIG(n = 34)
Neff G W 2004 [38]	USA	1994–2003	42	92	LAM(n = 51)	LAM+HBIG(n = 41)

*LAM, lamivudine; HBIG, hepatitis B immunoglobulin; ADV: adefovir dipivoxil; FTC,emtricitabine; TDF, tenofovir disoproxil fumarate

**Table 2:** Quality assessment of included studies.

Studies	End points	Follow-up period	Loss to follow up	control group	Contemporary groups	Baseline of groups	statistical analyses
Buti *et al.* [21]	A	A	A	A	A	A	A
Chun-Hui Yuan *et al.* [22]	A	A	A	A	A	A	A
Chung Mau Lo *et al.* [23]	A	A	A	A	A	B	A
Dai J *et al.* [24]	A	A	A	A	C	A	A
Dean M. Anselmo *et al.* [25]	A	A	A	A	C	A	A
Dennis A Freshwater *et al.* [12]	A	B	A	A	A	A	A
Lewis W. Teperman *et al.* [26]	A	A	A	A	A	A	A
Ma Y *et al.* [27]	A	A	A	A	C	A	A
Peter W. Angus *et al.* [28]	A	A	A	A	A	A	A
Shusen Zheng *et al.* [29]	A	A	A	A	A	B	A
Xia J *et al.* [30]	A	A	A	A	C	A	A
Xia N X *et al.* [31]	A	A	A	A	A	B	A
Xia Q *et al.* [32]	A	A	A	A	B	B	A
Yoshida H *et al.* [33]	A	A	A	A	C	A	A
Yuan GY *et al.* [34]	A	B	A	A	A	B	A
Jiao ZY *et al.* [35]	A	A	A	A	C	A	A
Zhu JP *et al.* [36]	A	A	A	A	C	A	A
Schiff *et al.* [37]	A	A	A	A	A	A	A
Neff G W *et al.* [38]	A	A	A	A	C	A	A

(1) End points: A, Endpoints appropriate to the aim of the study; B, no description about endpoints; C, endpoints inappropriate to the aim of the study. (2) Follow-up period: A, follow-up appropriate to the aim of the study; B, follow-up was not mentioned; C, follow-up in appropriate to the aim of the study. (3) Loss to follow-up: A, loss to follow-up less than 5%; B, no description about the loss; C, loss to follow-up over than 5%; (4) Control group: A, an adequate control group; B, no description about control group; (5) Contemporary groups: A, control and studied groups were managed during the same time period; B, no description about the time period of groups; C, control and studied groups were historical comparison. (6) Baseline of groups: A, groups were similar regarding the criteria other than the studied endpoints (like age, sex); B, no description about the baselines; C, baseline unequal among groups. (7) Statistical analyses: A, adequate statistical analyses; B, inadequate statistical analyses.

### HBV recurrence

**Figure 2:**
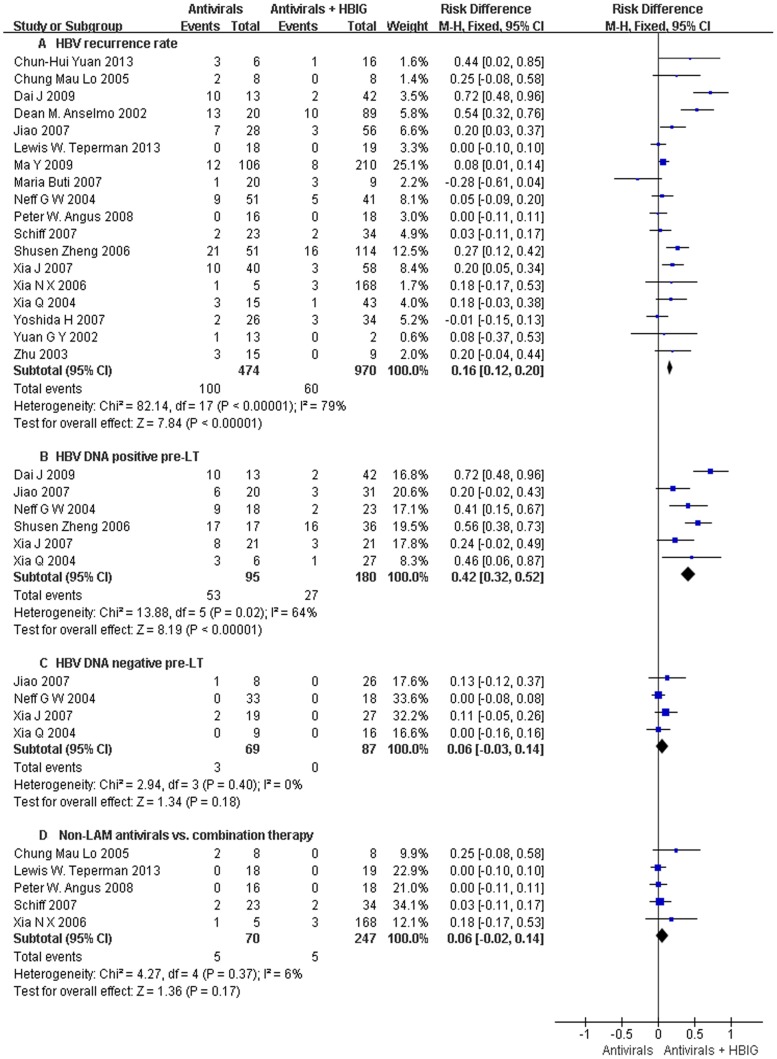
Antiviral drugs or antiviral drugs combined with HBIG in prophylaxis of hepatitis B recurrence after liver transplantation: results of a meta-analysis: HBV recurrence rate. (Columns represent the risk difference of each study. Diamonds represent the overall effect size, and diamond widths represent the overall 95% confidence interval.)

HBV recurrence was reported in 18 trials (Fig. 2.A.), and significant differences were observed between the two groups [P<0.001; RD = 0.16; 95% CI (0.12, 0.20)]. Combination of antiviral drugs and HBIG achieved a more favorable result to reduce the risk of HBV recurrence (Fig. 2.A.). In total, the HBV recurrence rates were 21.1% and 6.2% in antiviral drugs therapy group and combination therapy group respectively.

Subgroup analysis was conducted on patients with clarified pre-operative HBV-DNA status. Among all of the patients, 285 with positive HBV DNA before transplantation were included in 6 trials. It showed that antiviral drug therapy with HBIG was significantly more effective for HBV DNA positive patients [P<0.001; RD = 0.42; 95% CI (0.32, 0.52)] (Fig. 2.B.). Besides, 4 trials showed that in patients whose HBV DNA were negative before transplantation, there was no significant difference between the two therapies [P = 0.18; RD = 0.06; 95% CI (−0.03, 0.14)] (Fig. 2.C.).

Five trials used non-LAM therapies including ADV, ADV/LAM, and FTC/TDF (Fig. 2.D.). No significant difference was found between non-LAM therapy group and combination therapy group for HBV recurrence [P = 0.17; RD = 0.06; 95% CI (−0.02, 0.14)].

### YMDD mutants

**Figure 3:**
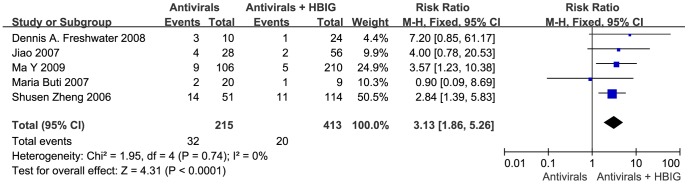
Antiviral drugs or antiviral drugs combined with HBIG in prophylaxis of hepatitis B recurrence after liver transplantation: results of a meta-analysis: YMDD mutants. (Columns represent the risk ratio of each study. Diamonds represent the overall effect size, and diamond widths represent the overall 95% confidence interval.)

YMDD mutants were reported in 5 trials (Fig. 3). Significant differences were observed in YMDD mutants rate between antiviral drugs therapy group and antiviral drugs combine with HBIG therapy group [P<0.001; RR = 3.13; 95% CI (1.86, 5.26)]. With the use of HBIG, patients showed a lower rate of YMDD mutants rate compared with antiviral drugs therapy.

### Patient survival

**Figure 4:**
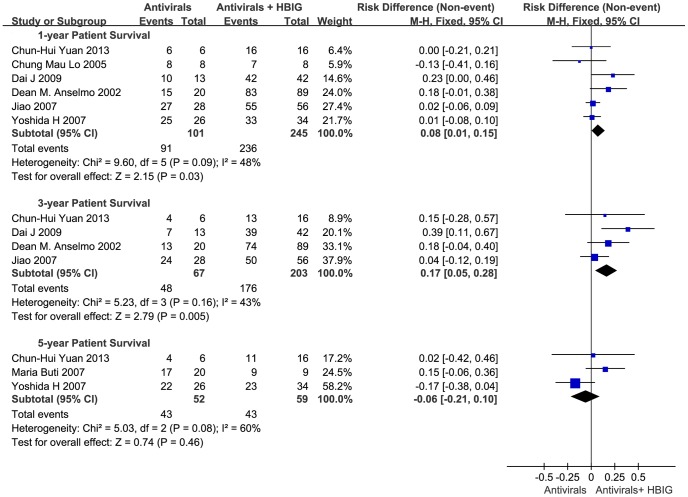
Antiviral drugs or antiviral drugs combined with HBIG in prophylaxis of hepatitis B recurrence after liver transplantation: results of a meta-analysis: 1-year/3-year/5-year patient survival rate. (Columns represent the risk difference of each study. Diamonds represent the overall effect size, and diamond widths represent the overall 95% confidence interval.)

One-year, 3-year and 5-year patient survival rate were analyzed (Fig. 4). Six trials calculated the 1-year patients' survival rate in 346 cases. 101 patients in antiviral drugs therapy group showed a 90.1% 1-year survival rate, and 245 patients in combination therapy group had a 96.3% 1-year survival rate. 3-year patient survival rates were counted in 4 reports involving 270 patients, which were 71.6% and 86.7% for patients in antiviral drugs therapy group and combination therapy group respectively. 111 patients' 5-year survival rates were reported in 3 trials. 82.7% patients in antiviral drugs therapy group survived longer than 5 years, while in combination therapy group, the 5-year survival rate was 72.9%.

Combination therapy achieved a higher 1-year and 3-year patient survival rate compared with antivirals therapy group [1-year survival P = 0.03; RD = 0.08; 95% CI (0.01, 0.15); 3-year survival P = 0.005; RD = 0.17; 95% CI (0.05, 0.28)]. However, there was no significant difference between the two groups in 5-year patient survival rate [P = 0.46; RD = −0.06; 95% CI (−0.21, 0.10)].

## Discussion

Application of nucleoside analogue has reduced the risk of Hepatitis B recurrence after LT dramatically. As the first generation of nucleoside analogue, LAM is safe and well-tolerated with efficacy against hepatitis B. Unfortunately, its long-term use is associated with viral resistance, which will lead to failure of HBV prophylaxis, and even the loss of the grafts and the patients. To reduce the risk of HBV mutating, variant doses of HBIG were introduced and have been very successful. In many transplant centers, prophylaxis with combined LAM and HBIG has been regarded as a routine procedure for such recipients.

The application of HBIG is associated with some disadvantages: inconvenience for patients to receive multiple injections of HBIG and surveillance of antibody titer. Also the cost would be an assignable burden economically. Some efforts have been made to verify the efficiency and safety of prophylaxis without HBIG and have achieved encouraging results [12], [26], [28], [38]. Fung J et al have also illustrated that long-term outcome using oral antiviral agents alone without hepatitis B immune globulin is associated with excellent survival [19]. While these results were not very convincing due to the lack of well-designed RCT and limitation of sample volumes.

Thus we conducted this meta-analysis to compare antiviral drugs therapy with combined antiviral drugs and HBIG therapy for the prophylaxis of hepatitis B recurrence after LT. We aim to clarify whether it is necessary to use HBIG in liver transplant recipients with HBV related diseases. Compared with previous studies, we also focused on the application of new nucleoside antiviral drugs and patients pre-transplant HBV-DNA status. Since we mainly compared between antiviral drugs therapy with or without long-term combination of HBIG, intro-operative usage of HBIG was not taken into consideration in our comparison. Antiviral drugs therapy following a period of combination therapy was also regarded as a monotherapy strategy [20], [23], [25], [29]. As shown in this meta-analysis, combination therapy has a significant advantage in terms of HBV recurrence and the virus mutation. It can also improve the 1 year and 3 year survival of the patients, although no significant improvement in patients' long-term survival has been observed.

Patients' pre-transplant virus replication status has been documented as the most important risk factor for virus recurrence and virus mutation; HBV DNA has always been regarded as a representative for HBV replication status [29], [35], [38]. In patients with negative pre-transplant HBV DNA, the risk of virus recurrence is much lower [35], [38]. Neff [38] reported that no additional advantage was conferred by combined use of LAM and HBIG compared with LAM monotherapy in patients with negative pre-transplant HBV DNA. In his study, 33 and 18 patients with negative HBV DNA received LAM alone and LAM+HBIG respectively had no HBV recurrence. Similarly, Xia J et al. [30], Xia Q et al. [32], and Saab et al. [39] also demonstrated that no evidence supporting the prophylactic use of HBIG+LAM combination therapy over LAM monotherapy in HBV DNA negative patients. We hypothesize that HBIG is not necessary in this group of patients. The subgroup analysis results in our meta-analysis are also consistent with the above conclusions. In our meta-analysis, HBV recurrence rate did not differ significantly between the two groups in patients with negative pre-transplant HBV DNA (P = 0.18, RD = 0.06 95%CI (−0.03, 0.14)). This result may be helpful for making a more reasonable HBV prophylaxis for patients after LT. In contrast, we found that patients with positive pre-transplant HBV DNA were at higher risk of HBV recurrence. In our study, 80 of the 275 included patients (29%) with detectable HBV DNA experienced HBV recurrence despite post-transplant antiviral therapy with a recurrence rate of 55.8% and 15% for antiviral drug therapy and combination therapy groups respectively. Combination therapy significantly reduced the HBV recurrence rate for those with positive HBV DNA before LT [P<0.001; RD = 0.42; 95% CI (0.32, 0.52)]. We conclude that the use of HBIG is necessary to decrease the risk of HBV recurrence in comparison to LAM monotherapy for liver recipients with positive HBV DNA before LT.

New nucleotide analogues including ADV and TDF have been proven effective and superior to LAM without risk of viral mutation [26], [28], [37], [40]. Some trials have also proven that maintenance therapy with newer nucleoside analogues after discontinuation of HBIG prophylaxis was effective [16], [41]. Based on their findings, we assumed that HBIG might be unnecessary during the post-transplantation period when the new analogues were used, and we performed a sub-group analysis of non-LAM antiviral versus combination therapy. As showed in our sub-group analysis, when using the new nucleoside analogues, there is no significant difference between the two groups for hepatitis B recurrence [P = 0.17; RD = 0.06; 95% CI (−0.02, 0.14)], but due to lack of patients' HBV-DNA status before LT and limited number of samples, this result is not compelling and remains to be determined. Patients' survival analysis showed that use of HBIG is beneficial in terms of 1-year and 3-year survival. And surprisingly, it showed no significant advantages for 5-year survival. Limited studies and small sample size might be an important reason for this result, in our analysis, only 3 studies evaluated 5-year survival rate, and there were only 111 patients in total.

There are several limitations to our study. Firstly, since LAM has been studied before, we had planned to evaluate the role of HBIG in such an era that new nucleoside drugs are used, while after an extensive literature searching, 14 of 19 included articles were about the use of LAM, among the other 5 studies adopted non-LAM antiviral drugs, ADV combined with LAM were used in 3, ADV monotherapy was used in 1, and FTC with TDF in 1 [22], [25], [27], [30], [36]. We included all of these researches and hopefully to get a convincing result on the basis of a large sample. Secondly, most of the included trials were not RCTs and 8 studies were retrospective studies. Thirdly, a few of included studies didn't set strict antiviral drugs therapy groups. For example, in Teperman's studies [25], they used a period of combination therapy before a comparison of antiviral drugs group with combination therapy. Aiming to evaluate the efficacy of HBIG in the long term prognosis after LT, we didn't exclude these literatures. The fourth limitation of our study is the lack of conditions of patients' pre-transplant HBV DNA. Nevertheless, we were able to draw a more convincing conclusion that the use of HBIG could be linked with patients' pre-transplant HBV DNA conditions.

In summary, this meta-analysis has found that HBIG is useful for prophylaxis of hepatitis B recurrence and YMDD mutants, but there is no significant difference between the antiviral drug therapy and combination of HBIG therapy with regard to patients' long-term survival. With the application of new nucleoside analogues, HBIG may be not necessary, especially in patients with negative HBV-DNA status before the operation. Well-designed RCTs with larger samples are still needed to evaluate the necessity of HBIG after LT.

## Supporting Information

Checklist S1 PRISMA Checklist.
(DOC)

## References

[1] Lai CL, Ratziu V, Yuen MF, Poynard T (2003) Viral hepatitis B. Lancet 362: 2089–2094. 14697813 10.1016/S0140-6736(03)15108-2

[2] Popescu I, Ionescu M, Brasoveanu V, Hrehoret D, Matei E, et al (2010) [Liver transplantation–indications, surgical technique, results–the analysis of a clinical series of 200 cases]. Chirurgia (Bucur) 105: 177–186. 20540229

[3] Freeman RB, Sanchez H, Lewis WD, Sherburne B, Dzik WH, et al (1991) Serologic and DNA follow-up data from HBsAg-positive patients treated with orthotopic liver transplantation. Transplantation 51: 793–797. 1901676 10.1097/00007890-199104000-00011

[4] O'Grady JG, Smith HM, Davies SE, Daniels HM, Donaldson PT, et al (1992) Hepatitis B virus reinfection after orthotopic liver transplantation. Serological and clinical implications. J Hepatol 14: 104–111. 1737910 10.1016/0168-8278(92)90138-f

[5] Samuel D (2009) The option of liver transplantation for hepatitis B: where are we? Dig Liver Dis 41 Suppl 2: S185–S189. 19482255 10.1016/S1590-8658(09)60442-4

[6] Starzl TE, Demetris AJ, Van Thiel D (1989) Liver transplantation (1). N Engl J Med 321: 1014–1022. 2674716 10.1056/NEJM198910123211505 PMC3091023

[7] Lo CM, Fan ST, Liu CL, Lai CL, Wong J (2003) Prophylaxis and treatment of recurrent hepatitis B after liver transplantation. Transplantation 75: S41–S44. 12589139 10.1097/01.TP.0000047027.68167.07

[8] Saab S, Kim M, Wright TL, Han SH, Martin P, et al (2000) Successful orthotopic liver transplantation for lamivudine-associated YMDD mutant hepatitis B virus. Gastroenterology 119: 1382–1384. 11054397 10.1053/gast.2000.19279

[9] Yang Y, Yang Y, Zhang J, Yi HM, Lu MQ, et al (2008) [Post-transplant prophylaxis of the recurrence of lamivudine-resistant YMDD mutant hepatitis B virus in liver recipients]. Nan Fang Yi Ke Da Xue Xue Bao 28: 1810–1812. 18971179

[10] Honaker MR, Shokouh-Amiri MH, Vera SR, Alloway RR, Grewal HP, et al (2002) Evolving experience of hepatitis B virus prophylaxis in liver transplantation. Transpl Infect Dis 4: 137–143. 12421458 10.1034/j.1399-3062.2002.01012.x

[11] Brock GN, Mostajabi F, Ferguson N, Carrubba CJ, Eng M, et al (2011) Prophylaxis against de novo hepatitis B for liver transplantation utilizing hep B core (+) donors: does hepatitis B immunoglobulin provide a survival advantage? Transpl Int 24: 570–581. 21401727 10.1111/j.1432-2277.2011.01236.x PMC3086971

[12] Freshwater DA, Dudley T, Cane P, Mutimer DJ (2008) Viral persistence after liver transplantation for hepatitis B virus: a cross-sectional study. Transplantation 85: 1105–1111. 18431229 10.1097/TP.0b013e31816a342a

[13] Rao W, Wu X, Xiu D (2009) Lamivudine or lamivudine combined with hepatitis B immunoglobulin in prophylaxis of hepatitis B recurrence after liver transplantation: a meta-analysis. Transpl Int 22: 387–394. 19017304 10.1111/j.1432-2277.2008.00784.x

[14] Yamamoto M, Little G, Imagawa DK (2009) Hepatitis B immunoglobulin in preventing reinfection following liver transplantation. Expert Rev Anti Infect Ther 7: 321–328. 19344245 10.1586/eri.09.2

[15] Fung J, Cheung C, Chan SC, Yuen MF, Chok KS, et al (2011) Entecavir monotherapy is effective in suppressing hepatitis B virus after liver transplantation. Gastroenterology 141: 1212–1219. 21762659 10.1053/j.gastro.2011.06.083

[16] Grellier L, Mutimer D, Ahmed M, Brown D, Burroughs AK, et al (1996) Lamivudine prophylaxis against reinfection in liver transplantation for hepatitis B cirrhosis. Lancet 348: 1212–1215. 8898039 10.1016/s0140-6736(96)04444-3

[17] Perrillo R (1999) Multicenter studies of Lamivudine for the treatment and prevention of hepatitis B after liver transplantation. Ochsner J 1: 33–36. 21845117 PMC3145427

[18] Wesdorp DJ, Knoester M, Braat AE, Coenraad MJ, Vossen AC, et al (2013) Nucleoside plus nucleotide analogs and cessation of hepatitis B immunoglobulin after liver transplantation in chronic hepatitis B is safe and effective. J Clin Virol 58: 67–73. 23880162 10.1016/j.jcv.2013.06.035

[19] Fung J, Chan SC, Cheung C, Yuen MF, Chok KS, et al (2013) Oral nucleoside/nucleotide analogs without hepatitis B immune globulin after liver transplantation for hepatitis B. Am J Gastroenterol 108: 942–948. 23629601 10.1038/ajg.2013.111

[20] Slim K, Nini E, Forestier D, Kwiatkowski F, Panis Y, et al (2003) Methodological index for non-randomized studies (minors): development and validation of a new instrument. ANZ J Surg 73: 712–716. 12956787 10.1046/j.1445-2197.2003.02748.x

[21] Buti M, Mas A, Prieto M, Casafont F, Gonzalez A, et al (2007) Adherence to Lamivudine after an early withdrawal of hepatitis B immune globulin plays an important role in the long-term prevention of hepatitis B virus recurrence. Transplantation 84: 650–654. 17876280 10.1097/01.tp.0000277289.23677.0a

[22] Yuan CH, Xiu DR, Jiang B, Li ZF, Li L, et al (2013) HBV recurrence lowered by lamivudine/HBIG combination therapy in liver transplant patients: ten-year experience. Hepatobiliary Pancreat Dis Int 12: 149–153. 23558068 10.1016/s1499-3872(13)60024-7

[23] Lo CM, Liu CL, Lau GK, Chan SC, Ng IO, et al (2005) Liver transplantation for chronic hepatitis B with lamivudine-resistant YMDD mutant using add-on adefovir dipivoxil plus lamivudine. Liver Transpl 11: 807–813. 15973721 10.1002/lt.20416

[24] Dai J, Lu S, Yan L, Li B, Lai W, et al (2009) [Long-term prevention of virus recurrence among recipients with HBV active replication following liver transplantation]. CHINESE JOURNAL OF HEPATOBILIARY SURGERY 15: 106–109.

[25] Anselmo DM, Ghobrial RM, Jung LC, Weaver M, Cao C, et al (2002) New era of liver transplantation for hepatitis B: a 17-year single-center experience. Ann Surg 235: 611–619, 619–620. 11981206 10.1097/00000658-200205000-00002 PMC1422486

[26] Teperman LW, Poordad F, Bzowej N, Martin P, Pungpapong S, et al (2013) Randomized trial of emtricitabine/tenofovir disoproxil fumarate after hepatitis B immunoglobulin withdrawal after liver transplantation. Liver Transpl 19: 594–601. 23447407 10.1002/lt.23628

[27] Yi MA, Qiang T, Xiao-shun HE, Guo-dong W, An-bin HU (2009) Prevention and management of hepatitis B virus reinfection after liver transplantation. CHINESE JOURNAL OF SURGERY 47: 1209–1212. 19781163

[28] Angus PW, Patterson SJ, Strasser SI, McCaughan GW, Gane E (2008) A randomized study of adefovir dipivoxil in place of HBIG in combination with lamivudine as post-liver transplantation hepatitis B prophylaxis. Hepatology 48: 1460–1466. 18925641 10.1002/hep.22524

[29] Zheng S, Chen Y, Liang T, Lu A, Wang W, et al (2006) Prevention of hepatitis B recurrence after liver transplantation using lamivudine or lamivudine combined with hepatitis B Immunoglobulin prophylaxis. Liver Transpl 12: 253–258. 16447195 10.1002/lt.20701

[30] Jie X, Zhan-yu Y, Jia C, Rui L, Nan Z, et al (2007) [Prevention and treatment of HBV reinfection after liver transplantation]. CHINESE JOURNAL OF DIGESTIVE SURGERY 6: 348–351.

[31] Xia N, Fu Z, Qiu B, Wang Z, Li X, et al (2006) [Low-dose intra-muscular hepatitis B immunoglobulin combined with lamivudine for long-term prophylaxis of hepatitis B recurrence after liver transplantation]. WORLD CHINESE JOURNAL OF DIGESTOLOGY 14: 1288–1293.

[32] Qiang X, Zhi-hai P, Jun L, Shu-yun W, Guo-qing C, et al (2004) [Low-dose hepatitis B immunoglobulin with Lamivudine in prophylaxis of hepatitis B recurrence after liver transplantation]. CHINESE JOURNAL OF ORGAN TRANSPLANTATION 25: 53.

[33] Yoshida H, Kato T, Levi DM, Regev A, Madariaga JR, et al (2007) Lamivudine monoprophylaxis for liver transplant recipients with non-replicating hepatitis B virus infection. Clin Transplant 21: 166–171. 17425740 10.1111/j.1399-0012.2006.00557.x

[34] Yuan G, Duan Y, Wang F, Liang SS, Zhu L (2002) [Prevention and treatment of HBV reinfection following liver transplantation]. Zhonghua Gan Zang Bing Za Zhi 10: 14–16. 11856491

[35] Jiao ZY, Jiao Z (2007) Prophylaxis of recurrent hepatitis B in Chinese patients after liver transplantation using lamivudine combined with hepatitis B immune globulin according to the titer of antibody to hepatitis B surface antigen. Transplant Proc 39: 1533–1536. 17580182 10.1016/j.transproceed.2007.03.062

[36] Zhu JP, Zhang TL, Li L, Yuan J, Song SB, et al (2003) Prevention and treatment of hepatitis B recurrence after liver transplantation. Hepatobiliary Pancreat Dis Int 2: 500–503. 14627508

[37] Schiff E, Lai CL, Hadziyannis S, Neuhaus P, Terrault N, et al (2007) Adefovir dipivoxil for wait-listed and post-liver transplantation patients with lamivudine-resistant hepatitis B: final long-term results. Liver Transpl 13: 349–360. 17326221 10.1002/lt.20981

[38] Neff GW, O'Brien CB, Nery J, Shire N, Montalbano M, et al (2004) Outcomes in liver transplant recipients with hepatitis B virus: resistance and recurrence patterns from a large transplant center over the last decade. Liver Transpl 10: 1372–1378. 15497163 10.1002/lt.20277

[39] Saab S, Waterman B, Chi AC, Tong MJ (2010) Comparison of different immunoprophylaxis regimens after liver transplantation with hepatitis B core antibody-positive donors: a systematic review. Liver Transpl 16: 300–307. 20209589 10.1002/lt.21998

[40] Wong TC, Fung JY, Lo CM (2013) Prevention of recurrent hepatitis B infection after liver transplantation. Hepatobiliary Pancreat Dis Int 12: 465–472. 24103275 10.1016/s1499-3872(13)60074-0

[41] Cholongitas E, Vasiliadis T, Antoniadis N, Goulis I, Papanikolaou V, et al (2012) Hepatitis B prophylaxis post liver transplantation with newer nucleos(t)ide analogues after hepatitis B immunoglobulin discontinuation. Transpl Infect Dis 14: 479–487. 22624695 10.1111/j.1399-3062.2012.00741.x

